# Microbiota–gut–brain axis: interplay between microbiota, barrier function and lymphatic system

**DOI:** 10.1080/19490976.2024.2387800

**Published:** 2024-08-25

**Authors:** Miaomiao Zhuang, Xun Zhang, Jun Cai

**Affiliations:** a Hypertension Center, Fuwai Hospital, State Key Laboratory of Cardiovascular Disease of China, National Center for Cardiovascular Diseases of China, Chinese Academy of Medical Sciences and Peking Union Medical College, Beijing, China; b Institute of Microbiology, Chinese Academy of Sciences, IMCAS, Beijing, China; c Beijing Anzhen Hospital, Capital Medical University, Beijing Institute of Heart, Lung and Blood Vessel Diseases, Beijing, China

**Keywords:** Microbiota-gut-brain-axis, lacteal, intestinal barrier, blood brain barrier, lymphatic system

## Abstract

The human gastrointestinal tract, boasting the most diverse microbial community, harbors approximately 100 trillion microorganisms comprising viruses, bacteria, fungi, and archaea. The profound genetic and metabolic capabilities of the gut microbiome underlie its involvement in nearly every facet of human biology, from health maintenance and development to aging and disease. Recent recognition of microbiota – gut – brain axis, referring to the bidirectional communication network between gut microbes and their host, has led to a surge in interdisciplinary research. This review begins with an overview of the current understandings regarding the influence of gut microbes on intestinal and blood-brain barrier integrity. Subsequently, we discuss the mechanisms of the microbiota – gut – brain axis, examining the role of gut microbiota-related neural transmission, metabolites, gut hormones and immunity. We propose the concept of microbiota-mediated multi-barrier modulation in the potential treatment in gastrointestinal and neurological disorders. Furthermore, the role of lymphatic network in the development and maintenance of barrier function is discussed, providing insights into lesser-known conduits of communication between the microbial ecosystem within the gut and the brain. In the final section, we conclude by describing the ongoing frontiers in understanding of the microbiota – gut – brain axis’s impact on human health and disease.

## Introduction

The human body hosts a rich ecosystem of microorganisms, such as bacteria, viruses, and fungi, collectively referred to as the microbiota.^1^ The human gastrointestinal (GI) tract contains trillions of microorganisms that exist symbiotically with the host due to a tolerant, regulatory cell- rich intestinal immune system. The central nervous system (CNS), comprising the brain and spinal cord, governs vital functions such as respiration, heart rate, movement, sensation, cognition, and reasoning, making it arguably the most crucial of all organ systems. The microbiota-gut-brain axis (MGBA) refers to the interaction between host microbiome, the CNS and the gastrointestinal tract.

Balance along the axis has implicated various pathways to maintain homeostasis including digestion, metabolism, barrier integrity, and immunity.^2^ Understanding the mechanisms of how gut microbiota community modulates the organ-specific environment remains research hotspot of present and future studies.

Barriers extending beyond the gut epithelial barrier, spanning the MGBA, are emerging as novel pathways facilitating communication between the gut microbiome and the brain. Disruption of the barrier integrity contributes a variety of gastrointestinal and neurological diseases. For decades, our understanding of barriers has shifted from perceiving them as rigidly impermeable cellular structures to dynamic and finely regulated communication interfaces with varying levels of permeability.

In this review, we explore barrier structure and function across the MGBA and examine the modulation of barrier function upon gut microbiota alteration. Additionally, we provide a summary of current knowledge concerning the lymphatic vasculature in the GI tract and CNS, highlighting its role in linking the reciprocal relationship between the lymphatic system and the microbiota, which collectively contributes to whole-body homeostasis. A thorough comprehension of the MGBA will foster the development of effective therapeutic interventions for the management of diseases.

## Gut microbiota and the intestinal barrier

The intestine primarily serves two key functions: the absorption of nutrients and the regulation of the transport of potentially harmful antigens and microorganisms. Functionally, intestinal microbiota plays a pivotal role in modulating intestinal barrier permeability and preserving the integrity of the enteric nervous system (ENS).^3,4^

### The gut barrier

**Figure 1.**
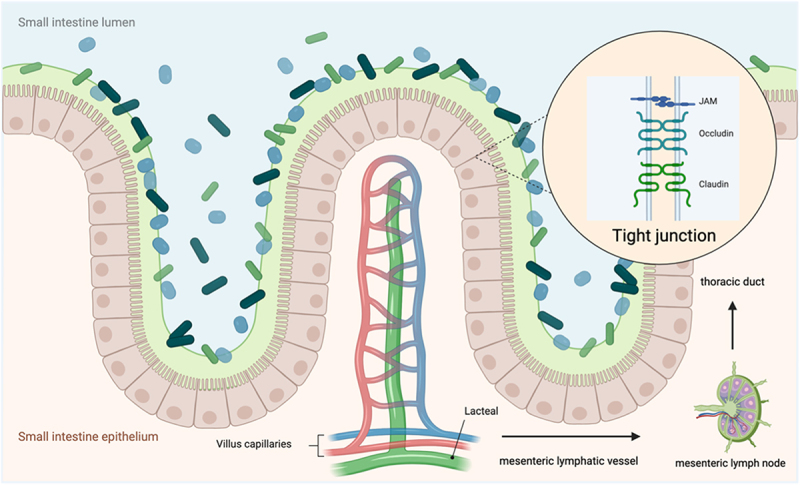
Illustration of gastrointestinal barrier. A layer of mucus, secreted by epithelial cells, coats the luminal side of the gut epithelium. The gut endothelial barrier comprises gut epithelial cells, tightly sealed by junctional complexes consisting of tight and adherens junctions. Within the gut epithelium are specialized cells, including enteroendocrine cells, which secrete gut hormones and serve as a crucial link in the communication between the central and enteric nervous systems. The gut vascular barrier is composed of fenestrated epithelium, also sealed by junctional complexes of tight and adherens junctions. Beneath the villus, intestinal lymphatic capillaries (lacteals) are situated, which drain into the mesenteric collecting vessels and the thoracic duct. This structure form a semipermissive barrier for lipid absorption and focal immune surveillance. This figure was created with BioRender (https://biorender.com/).

The term “gut barrier” refers to a physiological barrier system located within the intestines. It is primarily composed of the intestinal mucosa epithelium, mucous layer, and the intestinal immune system. The main function of the gut barrier is to maintain intestinal homeostasis, protecting the body from infections and preventing immune system dysregulation. Dysfunction of the gut barrier can lead to intestinal inflammation, autoimmune diseases, among other health issues.^5^ The protective function of the epithelial barrier occurs via three different layers (Figure 1).

The first layer of defense is the mucus secreted by goblet cells adherent to epithelial cells, the thickness of which increases along the length of the gut, mirroring the higher abundance of resident microorganisms. The mucus layer not only limits the exposure of epithelial cells from pathogens and the gut microbiota, also acting as a nutrient source and a colonization niche for the microbiota.^6^ Disruption of this layer is associated with initiation of inflammatory responses, leading to intestinal inflammatory disorders such as Crohn’s disease and ulcerative colitis.^7^ The second layer of defense is provided by the monolayer of tightly sealed epithelial cells that line the gut lumen. The gut epithelial barrier function is modulated by enteric glia, resembling the way astrocytes in neurovascular unit (NVU), indicating the revolutionary similarity of gastrointestinal and blood – brain barrier (BBB) function.^8^ The gut vascular barrier is the third layer of the intestinal barrier, which closely interacts with endothelial cells, glial cells and pericytes and regulates the translocation of intestinal content, such as bacteria, other microorganisms, toxins, proteins, bacterial metabolites, cytokines, immune and inflammatory cells, into the systemic circulation and, in turn, into organs remote from the intestine.^9^ The gut microbiota has been found to be involved in the development and preservation of the gut vascular barrier.^10–12^

Gut microbiota dysbiosis and leakage of bacterial products into the systemic circulation contribute to low-grade systemic inflammation.^13^ Metabolic endotoxemia and increased permeability of the intestinal barrier resulted from the systemic translocation of bacteria-derived lipopolysaccharides in metabolic syndrome are intricately regulated by shifts in the gut microbiota and could cause a systemic inflammatory cascade affecting multiple organs.^14,15^

A defective intestinal barrier is now considered the culprit of many gastrointestinal diseases including celiac disease and inflammatory bowel diseases, and of extra-intestinal disorders such as cirrhosis, and metabolic disorders, nonalcoholic steatohepatitis.^16–18^ Moreover, disruption of gut vascular barrier disruption due to an inflammatory insult induces closure of the choroid plexus vascular barrier in mice, suggesting a functional link between barriers along the gut – brain axis, which might be responsible for the commonly concurrent presentation of neurological and gastrointestinal symptoms.^19,20^

### Gut lymphatic vessel: new conduit in gut barrier

#### General organization of lymphatic system

The lymphatic system, running parallel to the blood circulation, is essential for maintaining fluid homeostasis, facilitating lipid absorption, and orchestrating immune responses in the body. It consists of an extensive network of lymphatic vessels that serve as conduits for transporting lymph fluid, immune cells, and various macromolecules. Interstitial fluid drains into blind‐ended, permeable lymphatic capillaries forming lymph; lymphatic collecting vessels transport lymph through lymph nodes to the thoracic duct and back into blood circulation.

Lymphatic vessel development follows a stepwise, highly conserved, and precisely regulated program mediated by biochemical and mechanical signaling pathways. These vessels gradually acquire specialized network characteristics in response to organ-specific environments. The intestinal lymphatic, responsible for transport of lipid absorbed by the intestinal mucosa and second line of defense against possible bacterial infections, is mutually regulated by signals of intestinal microbiota.

#### Intestinal lymphatic organization

The intestinal lymphatic system is composed of specialized vessels that transport lipids, lipid-soluble vitamins and hormones throughout the body. The initial lymphatics or lacteals consist of blind-ended capillaries, which are located in the center of villi, normally reaching 60–70% of the villus length, drain into the collecting lymphatics through intermediary or pre-collector lymphatic vessels.^21^ Water-soluble molecules enter blood vessels and are transported to the portal vein; conversely, lipids, macromolecules as well as potential pathogens enter lymphatic vessels, which then reach the blood circulatory system without passing through the liver.^22,23^

Intestinal lymphatic system is recently considered as a fourth layer of the intestinal barrier for the close association lacteals and microbiota exerts on the whole-body homeostasis. Much recent research has concentrated on identifying the primary sites where bacteria, bacterial-derived toxins, or tissue-derived toxins may potentially translocate to the lymphatic system, aiming to uncover potential intervention points at the initial stages of this potentially life-threatening process. Therefore, it is imperative to attain a comprehensive understanding of the influence of gut microbiota on the development and maintenance of lacteals. Meanwhile, knowledge regarding the two-way relationship between lymphatic function and gut microbiota remains an area requiring further investigation.

#### Crosstalk between microbiota and lacteals

The maturation of lacteals is governed by two distinct yet paradoxically opposing mechanisms. During development, lacteals undergo extension to increase the draining surface area exposed to the villus interstitial space, facilitating maximal permeability to accommodate the entry and passive diffusion of chylomicrons through the lacteals. Simultaneously, lacteals serve as a secondary line of defense against pathogens infiltrating the mucosal epithelium. According to the “lymph-based bacterial translocation” theory, submucosal dendritic cells containing captured bacteria are transported through lacteals to the first mesenteric lymph node before triggering an immune response, posing a potential risk for uncontrolled dissemination of pathogens through mesenteric lymphatics.^24–26^ Therefore a dynamic balance is reached between the final density and composition of host microbiota and the adult lacteals’ shape and functionality.

Dysfunction of the lymphatic network may lead to failure in immune homeostasis, resulting in alterations and translocation of microbiota, further exacerbating lymphatic vascular dysfunction and potentially leading to rapid lethality.^27–29^ Intestinal infection has been considered as pathogenesis in Crohn’s disease, and long-term changes in lymph flow and intestinal lymphatic function have been proposed as drivers of inflammatory bowel disease.^30^ Changes in composition and function of the gut microbiota are closely related to the progression of metabolic and chronic diseases.^31^ While increased lymphatic drainage by supplementation of Vascular Endothelial Growth Factor C (VEGFC) alters the host profile of intestinal microbiota, reducing chronic colitis in animal model, with an increased *Bacteroidetes/Firmicutes* ratio being observed.^32^

Moreover, the endothelial cell junctions are dynamic structures modulated by chylomicron uptake and intestinal bacteria, which has been proved in animal models with depletion of microbiota or antibiotic-treated mice.^33,34^ The extent of this effect varied by intestinal segment and correlated with the presence of microbiota, with the most significant changes observed in the jejunum and ileum, and the least in the duodenum, which is the least colonized segment of the intestine.^35^ Intestinal bacterial infection could profoundly remodel the intestinal environment by disrupting of lymphatic-mediated communication between tissues and the immune system. Acute infection of *Yersinia pseudotuberculosis* in the intestine leads to leakage of the mesenteric lymphatics in the mesenteric adipose tissue, compromising the tolerance and protective immune function in the canonical mucosal and mesenteric lymph node.^36^

## Gut microbiota and the brain barrier

The gut microbiota intricately influence brain development, function and behavior through neuroendocrine, immune pathways, and production of neuroactive substances, metabolites, and hormones in the blood and lymphatic system.^37^ While the host hormonal status, diet, lifestyle habits, and circadian cycle also dynamically alter the biodiversity of the gut microbiota.^38–41^ Though the gut and brain are anatomically separated, mounting evidence revealed that there is bidirectional communication between the gut microbiota and the brain.^42^

### The blood – brain barrier

Brain homeostasis relies on a tightly regulated and stable microenvironment to separate the CNS from the dynamic blood environment. Brain barriers serve to restrict paracellular diffusion into the brain while also acting as communication interfaces to receive peripheral circulating signals, including inputs from the microbiota. The blood – CNS barriers encompass two key interfaces: the BBB and the blood-cerebrospinal fluid (CSF) barrier (BCSFB). Additionally, the meningeal barrier, situated within the meninges, constitutes a further protective layer for the brain. The meninges consist of the pia mater, arachnoid mater, and dura mater.^43,44^ Notably, there are significant similarities between brain and gut barriers, both at the cellular and molecular levels, rendering them susceptible to modulation by common signals, including those originating from gut microorganisms.

The presence of dynamic and tightly regulated physiological changes in BBB function plays a key role in maintaining brain homeostasis in response to various environmental factor.^45^ The BBB is now recognized as an integral component of the NVU (Figure 1), which includes brain microvascular endothelial cells (BMEC), pericytes, astrocytes, neurons, microglia, and extracellular matrix.^46^ These organs, responsible for regulating the autonomic nervous system and endocrine glands, possess fenestrations that facilitate the diffusion of molecules across vessel wall.^47–49^ BMECs are linked through protein complexes comprising tightly packed, highly electrically resistant tight junctions. These junctions serve to restrict the paracellular movement of molecules between neighboring endothelial cells, thereby preserving ionic balance within the brain.^50^

The choroid plexus also constitutes an interface of exchange between the circulating blood and the CSF, which in turn contributes to the homeostasis of the extracellular fluid in the brain. The BCSFB consists of choroid plexus epithelial cells sealed by junctional complexes. The choroid plexus vascular barrier is composed of a fenestrated endothelium, constituting an interface of exchange between the circulating blood and the cerebral spinal fluid, which in turn contributes to the homeostasis of the extracellular fluid in the brain. The choroid plexus is also a reservoir of immune cells and soluble molecules, together with cerebral lymphatic vessels, acts as gateways for different signals into the brain.^48^

### Bidirectional pathways in microbiota-gut-brain axis

The gut microbiota communicates with the host in many ways, and host factors also influence gut microbiota composition and function. The brain modulates gut function through the hypothalamic-pituitary-adrenal axis and the autonomic nervous system. Conversely, the gut modulates CNS functions through involving a variety of microbiota-derived metabolites and products, neuroactive substances, and gut hormones that traverse through the enteric nervous system, vagus nerve (VN), circulatory system, or immune system to reach the brain. Collectively, these pathways are referred to as the MGBA, reflecting the evolutionarily ancient nature of this interdependent relationship.

#### Vagus nerve

The VN connecting the gut and the CNS represents the most direct conduit in the bidirectional communication between the gut microbiota and the brain, while mechanisms governing this intricate interplay are actively under investigation. The VN is the tenth cranial nerve and a crucial component of the parasympathetic nervous system.^51^ Functionally, the vagus nerve can detect mechanical and chemical stimuli within the gut and subsequently transmit the signal to the brain through afferent and efferent nerves, thus influencing both bottom-up and top-down signaling pathways.^52,53^ Vagal fibers with receptors for various microbiome-derived metabolites are capable of detecting alterations in microbial populations. Vagal terminals extending directly to the gut within the mucosal and smooth muscle layers can interact with enteroendocrine cells.^54^ Understanding the MGBA via the vagus nerve has paved the way for the exploration of novel treatment modalities for neuropsychiatric disorders.^55^ Intact VN is necessary to produce gut microbiota-induced effects. Experimental interventions with partial or complete vagotomy in rodents, result in alterations in brain circuits and behavioral functions, such as attenuation of gut microbiota-induced neurological phenotypes, mitigation in hippocampal inflammation, cognitive impairment alleviation.^56–60^ Likewise, direct stimulation of the VN regulates stress-induced depressive behaviors by modulating serotonergic circuitry in the hippocampus.^61,62^

#### Microbial-derived metabolites

Microbial-derived metabolites are major contributors in the MGBA that exert their effects primarily through receptor-mediated interactions on various host tissues or cells.^63,64^ The functionality of the MGBA is governed by an array of diverse neurotransmitters, neuropeptides, and microbial byproducts. Specifically, the metabolism of microbes on substrates like tryptophan, tyrosine exert a notable influence on the synthesis of pivotal neurotransmitters such as serotonin, noradrenaline, and dopamine.^65^ Evidence from studies suggests a potential contributory role of microbiota-generated neurotransmitters in modulating brain function.^66^

Short Chain Fatty Acids (SCFAs) are small organic monocarboxylic acids predominantly produced via colonic fermentation of dietary fiber and polysaccharides, serving as vital sources of energy and carbon for microbial proliferation.^67^ Beside the various physiological effects, including modulation of upper-gut motility, regulation of water and salt absorption, SCFAs can traverse the BBB directly, impacting its integrity.^68^ For instance, in germ-free mice, colonization with butyrate-producing bacteria or oral administration of sodium butyrate reduces BBB permeability by upregulating tight junction proteins.^63^ Furthermore, propionate treatment exhibits protective, anti-inflammatory, and BBB-permeability-reducing effects in a human brain endothelial cell culture model.^69^ Conversely, elevated levels of propionate in the brain may exacerbate symptoms in mouse models of autism, but supplementation with butyrate can mitigate these effects, suggesting the importance of metabolite balance.^70^ Trimethylamine N-oxide (TMAO), a microbial metabolite produced in foods such as eggs, nuts, dairy products, meat, and fish, has the ability to traverse the BBB and potential to restore the functionality of mutant tau protein, facilitating microtubule assembly.^71,72^ The detection of TMAO in human brains underscores the relevance of TMAO in Alzheimer’s disease (AD) pathology.^73^ Furthermore, certain strains of *Lactobacillus* and *Bifidobacterium* genera metabolize glutamate, the principal free amino acid and excitatory neurotransmitter in the brain, to yield γ-aminobutyric acid, a prominent inhibitory neurotransmitter.^74^

The interactions among microbial-derived metabolites and products within the MGBA exhibit considerable diversity and complexity: interacting with the intestinal epithelial barrier, the blood-brain barrier, directly influencing brain neurons, or modulating the endocrine and immune systems. A single metabolite has the potential to interact with multiple receptors across various tissue and cell types, thereby eliciting a wide range of physiological, immune, and CNS responses.^75^ These actions collectively contribute to shielding against the onset of pathology and inflammation linked to aging and disease.

#### Endocrine and immune signaling

Gut hormones, such as CCK, ghrelin, and 5-HT, are also important in gut-brain signaling and correlate with obesity and mood disorders via activating vagal sensory afferents.^76^ Microbiota is heavily implicated in the production and release of gut hormones while gut hormones may also affect the microbiota. For instance, germ free mice have significantly reduced levels of 5-HT and dopamine and promote GLP-1 secretion, compared to normal model.^77^ Meanwhile, 5-HT can be released toward the gut lumen, thereby altering the gut microbial profile.^78^ The gut microbiota plays important roles by interacting with mucosal immune system contributing to the surveillance of pathogens, tolerance of healthy gut ecology as well as maintaining the integrity of intestinal mucosal barrier to inhibit translocation of bacteria.^79,80^ The immune signaling in the MGBA regulates the release of mediators such as cytokines, neurotransmitters and neuropeptides, which modify the brain function through vagal nerve and other afferent fibers.^81^

### Crosstalk beyond organ and barrier in microbiota

Emerging evidence has reported the microbial colonization in accessory gastrointestinal organs and distant organs, such as the mouth, bladder, lungs and vagina, indicating a multi-organ crosstalk in microbiota balance and host behavior. The disrupted oral microbiota and microbe-derived metabolites have been associated with schizophrenia, suggesting a potential involvement of the oral-brain connection in the initiation of this disorder.^82–84^ The nasal microbiota plays a role in the MGBA by directly modulating the immune system, interacting with the olfactory system, and producing neurotransmitters or metabolites that traverse the BBB.^85^ Moreover, research also suggests crosstalk between the microbiomes of the gut and lung as well as the skin in neuropsychiatric disorders.^86–88^ Further research in this field is necessary to focus on the biological mechanisms, reliable biomarkers, and microbiota-targeted interventions in health and disease status.^85^

Barriers across the MGBA establish an interconnected system of epithelial and endothelial barriers that alter in brain barrier function to maintain homeostasis. Studies have shown the direct microbial invasion beyond the BBB while precise mechanisms remain incompletely understood.^89,90^ Bacteria may traverse the endothelium through either a transcellular pathway involving brain endothelial cells or a paracellular pathway by disrupting intercellular junctions.^91,92^ Increased bacterial population has been observed in Alzheimer brain tissue, suggesting that microbiological incursion into the CNS may significantly contribute to disease development.^93^ Research also found that fungus enters the brain from the blood by disrupting tight junction of the BBB, which could be eradicated by innate immune mechanism.^94^ Conversely, bypassing the BBB poses a significant clinical hurdle in drug delivery for treating neurological diseases. Therefore, comprehending how certain bacteria overcome CNS barriers through direct or indirect interactions is of clinical and therapeutical significance.

Given the considerable molecular and cellular similarities among barriers, it is probable that disruptions in barrier integrity associated with pathology occur across multiple barriers along the MGBA.

Recent research has implicated barrier function of MGBA in the pathogenesis of various neurological disorders, including neurodegenerative diseases, autoimmune diseases, neurodevelopmental and neuropsychiatric disorders, acute CNS injuries and spinal cord injury.^95–99^ Alterations in gut microbiota could contribute to barrier disruption at various levels: an altered microbiota entails changes in microbial-derived products, which could impact the function of gut and brain barriers; microbiota-disrupted barriers in the gut may become more permeable to microbial-derived products, subsequently allowing them to reach and potentially alter brain barriers; also, alterations in gut microbiota could influence barrier function through the modulation of gut and brain neuroimmune signals.

Further supporting the notion of inter-barrier communication across the MGBA, an influential study demonstrated that the choroid plexus vascular barrier closes upon gut vascular barrier opening associated with intestinal inflammation, which could be a mechanism to protect the brain from circulating inflammatory mediators.^20^ In addition, neonatal microbiota immaturity and incomplete intestinal and choroid plexus favor certain harmful bacteria colonization, translocation across the gut-vascular barrier, causing bacteremia and susceptibility to meningitis.^100^ Further research is needed to explore the potential of microbial signals in modulating transport across gut and brain barriers.

## Lymphatic network: new routes of gut microbiota to the brain

For decades, blood vessels and nerves were thought to be the primary pathways by which metabolites and toxins affect distant organs. It now appears that intestinal lymphatics constitute an additional pathway in the gut-organ axis. Although the brain parenchyma is devoid of lymphatic vessels, the rapid clearance of cellular debris and metabolic products in the CNS is attributable to the glymphatic system and meningeal lymphatic vessels.^101^ In the glymphatic system, cerebrospinal fluid flows into the brain through the para-arterial space, transports excess lipids, and drains into the cerebrospinal fluid circulatory system or directly through the capillary lymphatics into the cervical lymphatics. In more recent studies, meningeal lymphatic vessels were shown to be functionally linked to the glymphatic pathway, draining the cerebrospinal fluid into the cervical lymph nodes and involving in the elimination of molecules, cellular debris and waste products.^102^ Besides, meningeal lymphatic vessels are intersectional routes for the CNS and immune system, delivering antigen as well as immune cells to the cervical lymph nodes for CNS immune surveillance.^103^ Recent works on lymphatic vessels in the brain suggesting a possible connection to the body’s immune system and inflammation in CNS diseases^104–106^ (Table 1).

**Table 1.** Lymphatic function in neurological disorder.

Neurological Disease	Involvement of Lymphatics	Disease phenotype	references
Alzheimer’s Disease	Involvement in waste clearance from the brain; potential role in pathology and therapeutics.	Dysfunctional lymphatic pathways contribute to the accumulation of toxic proteins and disease progression. Therapeutic strategies targeting lymphatic function hold promise in alleviating pathology and decelerating disease advancement.	^102,107,108^
Parkinson’s Disease	Accumulation of alpha-synuclein in meningeal lymphatics; potential role in disease propagation.	Enlarged deep cervical lymph nodes (dCLNs) and reduced perfusion wete observed, attributed to increased macrophage activation, along with exacerbated motor and sensory dysfunction.	^109,110^
Aging	Decreased lymphatic function with age, impaired drainage of waste and immune cells.	Dural T cells increase toward non-sinus regions and exhibit enhanced IFN-γ expression in aging mice. Age-associated B cells from the blood accumulate in the dura, displaying an antigen-experienced pattern and differentiating into plasma cells.	^111,112^
Experimental Autoimmune Encephalomyelitis (EAE)	Inflammatory processes and immune surveillance within the CNS.	Bone marrow-derived myeloid cells in the dura infiltrate the CNS parenchyma during EAE, showing regulatory phenotypes. Disease- and tissue-specific transcriptional changes were observed in leptomeningeal myeloid populations in EAE mice.	^113–116^
Multiple Sclerosis (MS)	Inflammation-induced dysfunction of meningeal lymphatics; impaired drainage of cerebrospinal fluid.	Inflammation disrupts the normal functioning of meningeal lymphatics, impairing the drainage of CSF and metabolic waste from the CNS and contributing to the accumulation of inflammatory mediators and neurotoxic substances, exacerbating neuroinflammation and neuronal damage.	^117–119^
Traumatic Brain Injury and Stroke	Subarachnoid clot clearance, and erythrocyte drainage to dCLNs; activation of meningeal lymphatics post-injury; involvement in immune response and debris clearance.	Meningeal lymphatic activation and expansion participates in the clearance of immune cells, debris, and fluid from the CNS after stroke. Strategies aimed at modulating lymphatic function to enhance lymphatic drainage could reduce intracerebral hematoma volume, promote neuroprotection, tissue repair, and functional recovery post-stroke.	^120–123^

Dysfunction in meningeal lymphatic vessels protein clearance potentially contributes to the onset and aggravation of AD pathology.^124^ VEGFC-mediated lymphangiogenesis is capable of enhancing the immune response by recruiting T cell infiltration, inducing a long-lasting memory response in diseases such as glioblastoma and spinal cord injury.^125,126^ While the serious inflammatory response induced by meningeal lymphatic vessels may potentially enhance autoimmune neuroinflammatory conditions like multiple sclerosis, thus blocking this draining route seems to be an effective intervention practice.^127^

**Figure 2.**
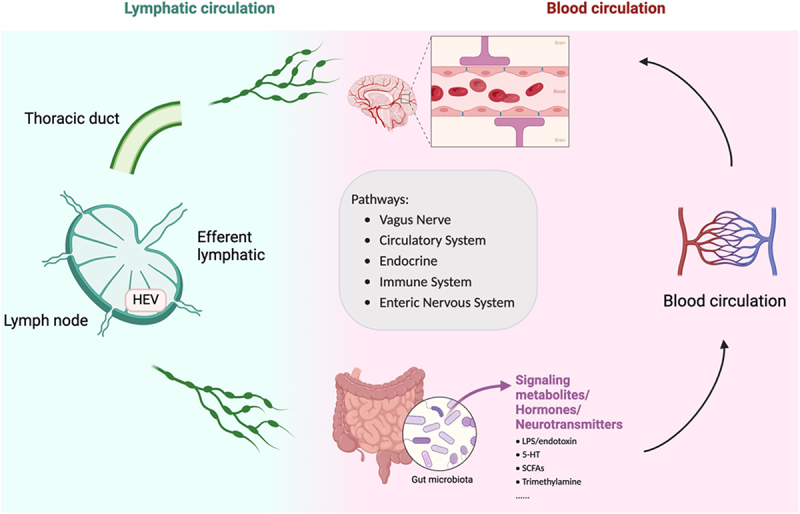
The microbiota-gut-brain axis. The bidirectional communication between the brain and gut microbiota is mediated by several pathways including the immune system, neuroendocrine system, ENS, circulatory system, and vagus nerve. Microbiota can promote production of essential metabolites, neurotransmitters, and other neuroactive compounds that influence the gut epithelial barrier and brain barrier. Different types of microbial metabolites have been shown to modulate barrier function by blood circulation (arrows). Alterations in gut microbiota have been linked to the development of autism spectrum disorders, anxiety, depressive-like behavior, impaired physical performance, and motivation, as well as neurodegenerative diseases. Recent research also suggesting the potential for microbiota to traverse the lymphatic system, serving as a secret pathway, linking the gut and the brain, may contribute to the development of disease. This figure was created with BioRender (https://biorender.com/).

The lymphatic network connects the MGBA impacting immune cells or metabolites that are transported to CNS area. Lymphatic network that links CNS with draining lymph nodes and enables bacterial-metabolite-associated neurotransmission would be an exciting frontier in clinical and experimental medicine. Numerous diseases are associated with deranged blood vessel endothelial barrier function, increased permeability, and extravasation into the microenvironment surrounding lymphatic vessels. It is conceivable that the microbiota might exert its effect on the initiation or progression of CNS disease through lymphatic network in a direct or indirect manner (Figure 2).

## Conclusions

The MGBA serves as a significant bidirectional communication network, presenting an actionable target for mitigating the development and progression in gastrointestinal and neurological diseases. Intestinal barriers and BBB constitute dynamic and adaptive structures crucial for facilitating key aspects of communication. Dysbiosis compromises the integrity of both the intestinal barrier and BBB, with recent evidence implicating the meningeal barrier as well. We explore the structural and functional similarities among barriers, while acknowledging their individual peculiarities crucial for their respective functions and cross-barrier communication. Notably, we discuss epithelial and vascular barriers, emphasizing their close interaction and remarkable similarities in barrier structure.

Mounting evidence supports the role of MGBA as a significant bidirectional communication network, presenting an actionable target for mitigating the development and progression in gastrointestinal and neurological diseases. Such observations foster collaboration among traditionally segregated fields of microbiology, immunology, lymphatics, and neuroscience, facilitating interdisciplinary research.

In clinical settings, prebiotics, fecal microbiota transplantation, and modulation in lymphatics are currently under active investigation for optimizing treatment selection to improve clinical prognosis. Nevertheless, critical questions and uncertainties remain unanswered regarding the MGBA, and limitations of preclinical models continue to obscure translational insights. Firstly, the wide variation in gut microbial profiles between individuals poses challenges as individual microbes may exhibit dual roles, capable of both benefiting and harming human health. Secondly, preclinical findings of existing animal models are primarily observed in rodent models and hindering successful bench-to-bedside translation. Appropriate animal models, coupled with advanced technologies like single-cell sequencing and computational methodologies, are required to capitalize on the potential of manipulating the MGBA in disease. Understanding the role of this co-evolution of hosts and their microbiota provides new therapeutic insights in field of gastrointestinal, neurodevelopmental, neurodegenerative, and cerebrovascular diseases.
